# Splanchnic venous thrombosis in *JAK2* V617F mutation positive myeloproliferative neoplasms – long term follow-up of a regional case series

**DOI:** 10.1186/s12959-018-0187-z

**Published:** 2018-12-19

**Authors:** Graeme Greenfield, Mary Frances McMullin

**Affiliations:** 1 Centre for Cancer Research and Cell Biology, Queen’s University Belfast, University Rd, Belfast, BT7 1NN Northern Ireland; 2 Centre for Medical Education, Queen’s University Belfast, University Rd, Belfast, BT7 1NN Northern Ireland

## Abstract

**Background:**

Splanchnic Vein Thrombosis (SVT) is strongly associated with underlying *JAK2* V617F positive myeloproliferative neoplasms (MPN).

**Methods:**

Patients attending the tertiary haematology service in Northern Ireland with SVT and underlying *JAK2* V617F MPN were identified by consultant staff. A retrospective audit was undertaken to examine therapeutic interventions and relevant outcomes. Descriptive statistics were used for qualitative data whilst students t-test allowed comparison of quantitative data.

**Results:**

We report on the medium to long term follow-up of fourteen patients presenting with SVT on the basis of *JAK2* V617F positive MPN. Females comprised 78.5% of the patients and there was an average age of 47.3 years at time of diagnosis. There was significant morbidity evident at diagnosis with liver transplantation attempted in all patients with Budd Chiari (*n* = 3), oesophageal varices present in 57.1%, ascites present in 42.8% and splenomegaly evident in 71.4%. 42.8% of patients did not exhibit classical phenotypic blood count findings for MPN at time of diagnosis. Over a median follow-up of 88.5 months (range = 8–211 months) recurrence of SVT was only documented in the setting of interventional liver procedure. Major haemorrhagic complications were recorded in 35.7% of patients and there was an association with dual anticoagulation and antiplatelet use. Recurrent thrombosis outside of the splanchnic venous system occurred in 28.5% of patients, predominantly occurring off therapeutic anticoagulation. No deaths were recorded and one transformation to myelofibrosis was seen during follow-up. Cytoreduction therapies were routinely used but had a high discontinuation rate due to cytopenias and intolerance.

**Conclusion:**

This analysis highlights the complexities of management of this group of patients over a period of long follow-up with a focus on the evidence behind therapeutic options.

## Background

Splanchnic vein thrombosis (SVT) describes a heterogeneous group of disorders involving venous thrombus within the portal vein (PVT), superior mesenteric vein, splenic vein or hepatic veins (Budd-Chiari Syndrome (BCS)). This life changing diagnosis is associated with significant morbidity resulting from liver decompensation. The Philadelphia chromosome negative myeloproliferative neoplasms (MPN) likewise compromise a heterogeneous group of clonal neoplastic disorders characterised by the overproduction of erythrocytes in polycythaemia vera (PV), platelets in essential thrombocythaemia (ET) and bone marrow fibrosis in myelofibrosis (PMF). The *JAK*2 V617F mutation identified in 95% of PV patients and around 50% of ET and PMF cases acts as a driver for clonal proliferation through constitutive activation of the JAK/STAT pathway [1]. *CALR* and *MPL* mutations are present as driver mutations in the majority of remaining cases [2, 3].

The presence of a *JAK2* V617F mutant clone predisposes to the formation of thrombus. This has been documented in essential thrombocythaemia where *JAK2* V617F positive patients have a higher rate of thrombosis and in clonal haematopoiesis of indeterminate potential where the presence of mutated *JAK2* is associated with an increased risk of coronary artery disease [4, 5]. *JAK2* V617F positive MPNs have been identified as the most common underlying cause in splanchnic vein thrombosis not associated with local factors, for example cirrhosis or malignancy. In a prospective study of 604 patients with proven SVT, underlying overt MPN was identified in 8% of patients whilst the *JAK2* V617F mutation was detected in 20% [6]. In a meta-analysis, it was demonstrated that 40.9% of SVT and 41.1% of BCS have an underlying MPN and 41.1% of PVT and 27.7% of BCS patients have the *JAK2* V617F mutation [7]. *JAK2* V617F allele burden is often low in these patients [8]. A small number of *JAK2* V617F negative patients will develop positivity with follow-up over subsequent months [9]. The number of *CALR* positive patients detected in the context of SVT is much lower at around 0–2.5% [10].

There is little published on the medium to long term outcomes of this specific patient population. This retrospective analysis of a case-series of 14 patients presenting with SVT on the basis of underlying *JAK2* V617F positive MPN was undertaken to examine these outcomes and response to real world therapy of this patient group. It highlights a number of key complexities in the management.

## Methods

Fourteen patients with SVT in the setting of *JAK2* V617F positive MPN were identified following attendance at the outpatient haematology service. A retrospective audit of therapeutic interventions and outcomes was undertaken. Electronic care records and patient notes were reviewed. Statistical analysis was performed using the Student’s t test for quantitative data. *P* values < 0.05 were considered statistically significant.

## Results

The relevant demographics of the patient population at presentation are shown in Table 1. In all cases, SVT was the presenting feature of MPN. All patients were positive for the *JAK2* V617F mutation as a condition of inclusion in this case series. Patients with MPN diagnosed prior to the discovery of the *JAK2* V617F mutation have been included on the basis of subsequent proof of positivity. Female patients comprised 78.5% and there was an average age of 47.3 years at the time of diagnosis across the cohort. Previous thrombotic events had been documented in two patients, these were one cerebral venous thrombosis and one placental thrombosis. In the case of the cerebral venous thrombosis this patient was on anticoagulation at the time of the SVT. No additional inherited or acquired pro-thrombotic conditions were detected however screening for these conditions was not universally performed (50% screened for PNH, 43% for Factor V Leiden, 71% for antiphospholipid syndrome, 36% for anti-thrombin deficiency and 29% for protein C or protein S deficiency). There was a very low incidence of co-existing cardiovascular risk factors and/or hormonal therapy use.

**Table 1:** Patient Demographics

	Budd Chiari		Portal Vein Thrombosis Only		Portal Vein Thrombosis +/− Splenic Vein +/− Superior Mesenteric Vein	
Number	3		7		4	
Sex	Male	0	Male	3	Male	0
	Female	3	Female	4	Female	4
Age	Mean	38.6	Mean	47.5	Mean	53.5
	Range	22–49	Range	36–67	Range	38–84
Type of MPN						
PV	1		2		2	
ET	2		1		0	
PMF	0		0		0	
Latent/Unclassifiable	0		4		2	
Blood Counts						
Hb (g/l)	Mean	136	Mean	133^a^	Mean	140
	Range	127-145	Range	93–200	Range	111–179
White Cell Count (× 10^9^/l)	Mean	16.6	Mean	9.4^a^	Mean	11.6
	Range	11.7–21.3	Range	5.1–14	Range	8.3–17
Platelets (×10^9^/l)	Mean	592	Mean	358^a^	Mean	537
	Range	539-656	Range	283–649	Range	369–861
Haematocrit	Mean	0.62^a^	Mean	0.40^a^	Mean	0.41
	Range	0.39–0.46	Range	0.31–0.57	Range	0.33–0.54
Other Inherited pro-thrombotic condition	0		0		0	
On Anticoagulant at time event	1		0		0	
On Anti-platelet at time of event	0		0		0	
Other cardiovascular risk factors	0		1		0	
On OCP/HRT at time of event	1		0		0	
Previous Venous Thrombosis	1		1		0	
Previous Arterial Thrombosis	0		0		0	
Prior features suggestive MPN	1		3		0	
Liver Intervention						
Initial TIPPS	2		0		0	
Liver Transplant	2		0		0	
Failed Transplant	1		0		0	
Complications						
Ascites	3		1		2	
Splenomegaly	2		5		3	
Varices	0		6		2	
Follow-up (months)	Mean	114	Mean	116	Mean	50.7
	Range	47–211	Range	65–204	Range	8–118

This shows the demographics of the patient population in each subclassification of splanchnic vein thrombosis

^a^Data not available for 1 patient

Significant morbidity from liver failure were evident at diagnosis. Portal venous hypertension was common in all groups of SVT with 42.8% of patients presenting with ascites and 57.1% of patients presenting with oesophageal varices. Splenomegaly was particularly common, evident in 71.4% of patients and may reflect the potential dual aetiology of MPN and portal venous hypertension. Liver interventions were common in the Budd Chiari patient group with all three patients undergoing attempted transplantation. Of these attempts, one was abandoned during the procedure and the other two were successful. Trans-jugular intrahepatic portosystemic shunts (TIPS) had been used prior to transplant in two of these patients. There were no invasive liver procedures in the non Budd Chiari group other than banding of varices. One patient has developed definite cirrhosis during follow-up confirmed on fibroscan, this was the one Budd Chiari patient who had a failed liver transplant.

In six of the fourteen patients (42.8%), the initial full blood count was not typical of a classical myeloproliferative neoplasm and did not meet diagnostic criteria for classification as PV, ET or PMF. This finding of a high number of “latent” MPN diagnoses is commonly reported in this group.

Median follow-up was 88.5 months (range = 8–211 months) with no deaths recorded during this time. Transformation to secondary myelofibrosis was recorded in one patient at 118 months follow-up. There were no recorded transformations to acute leukaemia. One patient was lost to follow-up at 96 months due to relocation.

**Fig. 1:**
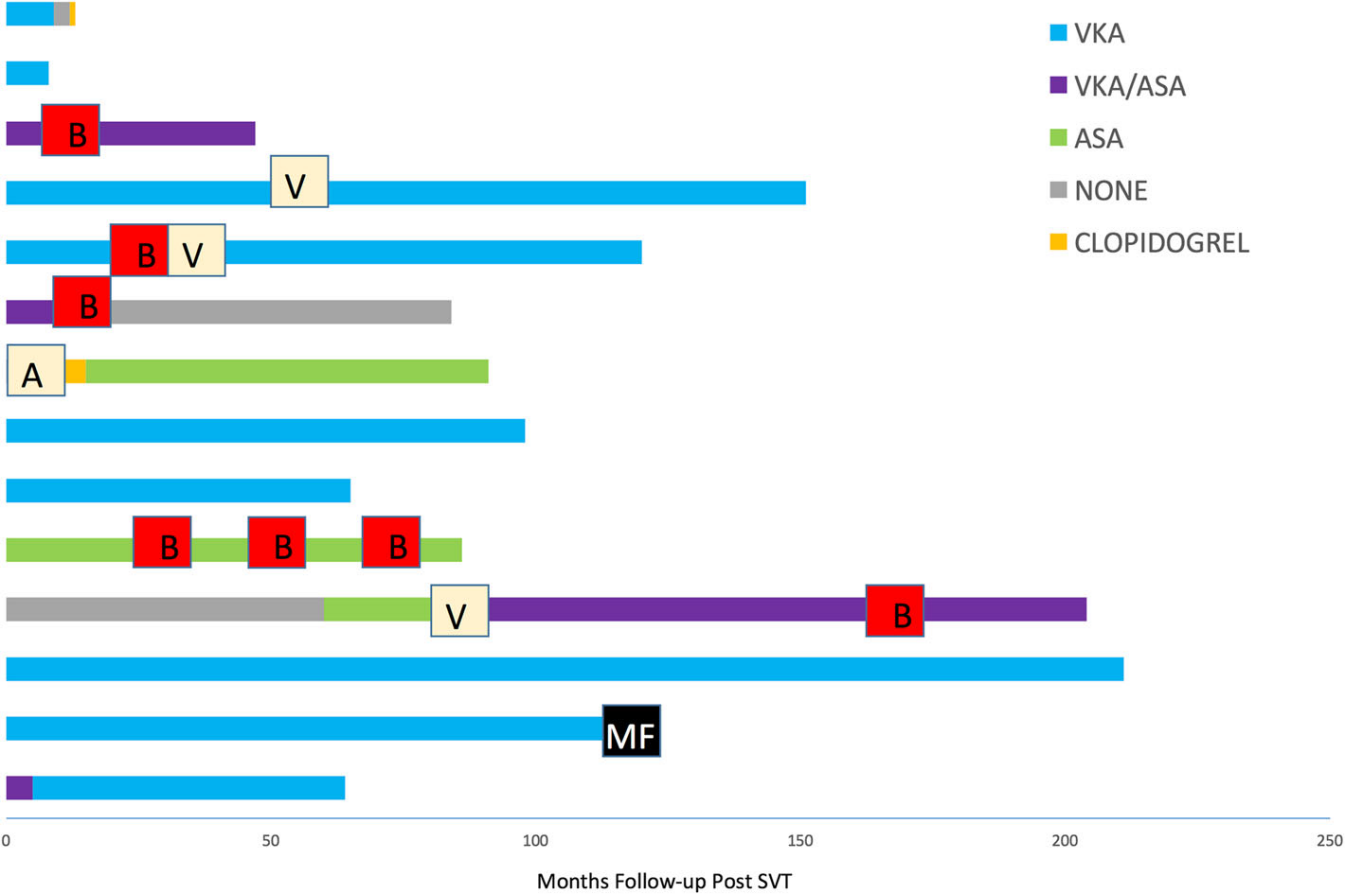
A diagram showing the use of anticoagulation and/or antiplatelet agents in the management of each patient. Each bar shows time of follow-up each therapy was used from point of initial diagnosis through to most recent follow-up. Episodes of Bleeding (B) are marked with a red box, Episodes of Arterial (A) and Venous (V) thrombosis are marked with a cream box and transformation to Myelofibrosis (MF) is marked with a black box. VKA = Vitamin K antagonist, ASA = Aspirin, VKA/ASA = Dual Vitamin K antagonist and Aspirin therapy

The complexity of management was demonstrated by the variation in anticoagulation and antiplatelet therapeutic strategies employed at initial diagnosis through to recent follow-up. Figure 1 demonstrates the use of anti-thrombotic strategies in each patient. After diagnosis, the majority of patients were managed on single agent anticoagulation with Vitamin K antagonist (VKA) therapy. A smaller number received dual therapy with VKA alongside an aspirin. However, two of these three patients had this strategy discontinued relatively quickly in the course of follow-up and only one additional patient was commenced on this approach following a further thrombotic event. Antiplatelet only regimes were less frequently used. One patient was withdrawn from either anti-coagulation or anti-platelets due to recurrent bleeding. Low molecular weight heparins were only used on short term basis initially or as bridging therapy, novel oral anticoagulants were not used.

**Fig. 2:**
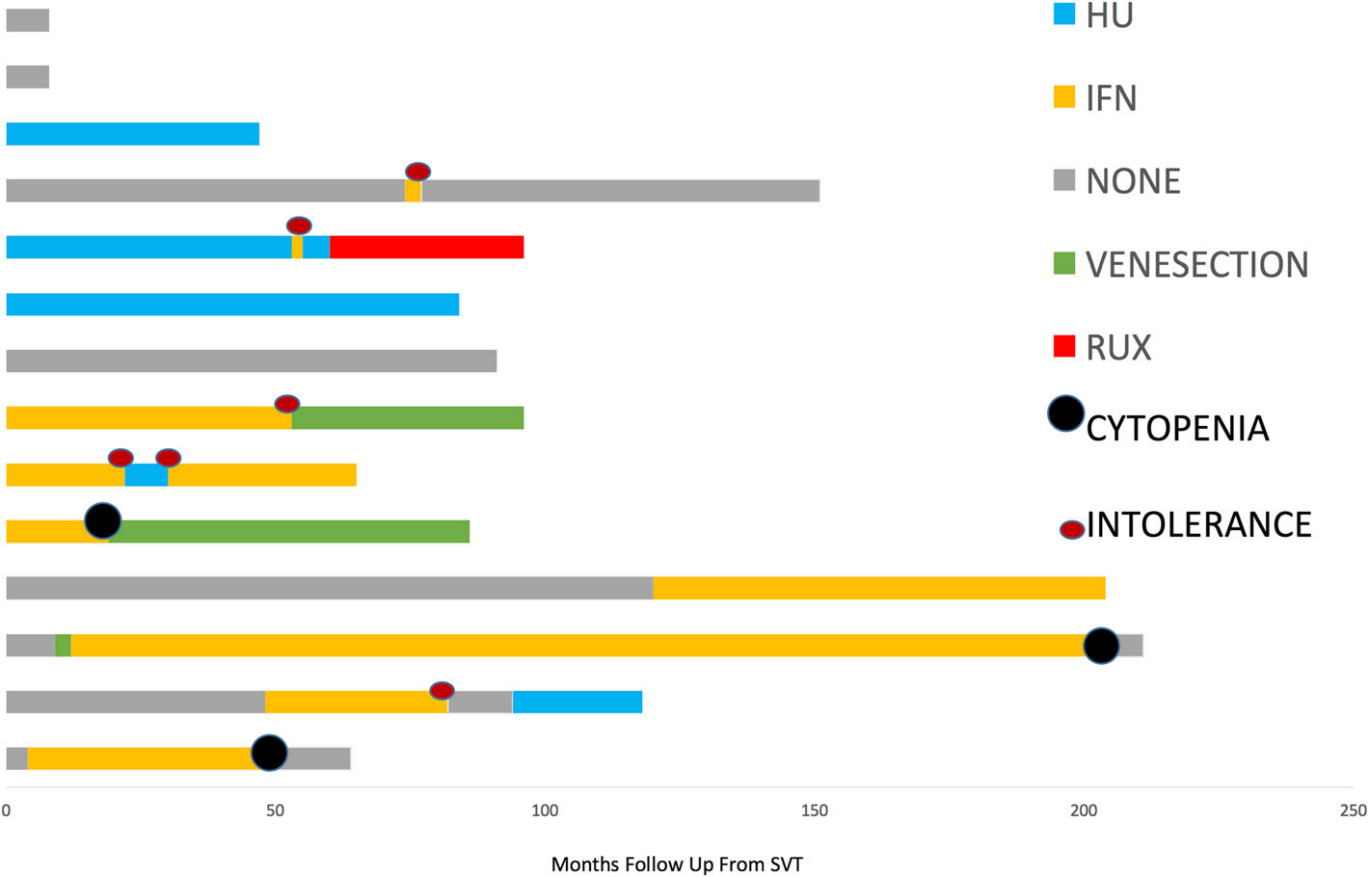
A diagram demonstrating the cytoreductive therapies used in the management of each patient. Each bar shows the time from point of diagnosis through to most recent follow-up that each therapy was used. Episodes of Cytopenia and Intolerance are recorded. HU = Hydroxycarbamide, IFN = Interferon, RUX = Ruxolitinib

Hydroxycarbamide and interferon alpha are the most common agents used for cytoreduction. Use of these agents was widespread with 78.5% of patients exposed to cytoreduction during follow-up. Rates of intolerance and ineffectiveness of therapy were high with a number of changes in the therapeutic strategy used evident during follow-up. Reasons for discontinuation of therapy included medication side effects, cytopenias, ineffectiveness and clinical trial participation. Figure 2 demonstrates the use of cytoreduction. Reasons for change of therapy are recorded. From point of diagnosis through to most recent follow-up, the use of cytoreductive therapies across the cohort resulted in a significant decrease in the total white cell count with a decrease of 4.4 × 10^9^/l (*P* = 0.004) and platelets with a decrease of 219 × 10^9^/l (*P* = 0.001) and a non significant reduction in haemoglobin. This is consistent with the known actions of these drugs.

In half of patients, follow-up was complicated by thrombosis, bleeding or both. The time points for these events are demonstrated in Fig. 1. Significant haemorrhagic events recorded during follow-up were oesophageal variceal bleeds, haematuria, tongue laceration and sub-dural haematoma. Recurrent variceal haemorrhage occurred in one patient. Of the bleeding events recorded, patients were on dual anticoagulation and antiplatelet therapy in three cases, antiplatelet therapy only in one case and anticoagulant therapy only in one case demonstrating a particularly high bleeding risk with dual therapy.

Recurrence of SVT only occurred once during an invasive liver procedure (attempted liver transplantation) in a BCS patient. A TIPS shunt was occluded in one patient. Outside of the splanchnic venous system, there was one arterial event (transient ischaemic attack) during follow-up and three venous thrombotic events. These were bilateral pulmonary emboli, cerebral venous thrombosis and bilateral jugular vein thrombosis. One of these events occurred on therapeutic anticoagulation, one on prophylactic low molecular weight heparin and one on aspirin.

## Discussion

Our case series documents the medium to long term follow-up for patients with SVT and underlying *JAK2* V617F positive MPN, highlighting a number of complexities in the management of these patients. Our findings were in keeping with previously documented observations that the demographic of this group tends towards younger, female patients [8]. This contrasts to the proven high thrombotic risk group within the general MPN population which is defined by age over 60 years and previous thrombosis [11]. Unsurprisingly, we noted significant morbidities at the outset for all groups of SVT, with liver transplant attempted in all BCS patients. The presence of the *JAK2* V617F clone has been associated with higher Child-Pugh scores in BCS indicative of more severe pathology [12].

Current recommendations suggest that the absence of classical blood count findings in unprovoked SVT should not preclude *JAK2* V617F testing [7]. Our findings would again emphasize this importance with almost half of the patients presenting without a classical MPN phenotype in the full blood count. A number of reasons have been suggested to account for this including hypersplenism, haemodilution related to portal hypertension and/or iron deficiency related to blood loss. Elevated red cell masses have been demonstrated in this cohort despite normal haematocrit [12]. The presence of *JAK2* V617F appears to function as an all or nothing effect with *JAK2* V617F allele burden previously observed to be low in this patient group [8] The number of reported cases of SVT in the presence of *CALR* or *MPL* mutations are very low [13]. This is in keeping with a direct role of the presence of the *JAK2* V617F mutation on the propensity to form clot in the splanchnic veins. There is evidence to suggest increased interaction between PV erythrocytes and endothelial cells resulting from increased phosphorylation of glycoproteins mediated directly by abnormal JAK/STAT signalling [14]. The *JAK2* mutation has also been detected in the endothelial cells of the liver in BCS patients [15], endothelial progenitor cells [16] and splenic endothelial cells [17] therefore allowing the hypothesis that abnormal, activated blood cells interact with abnormal endothelial cells resulting in a propensity to form clot. Of interest, *JAK2* V617F mutations have been identified in individuals with no MPN phenotype. This “clonal haematopoiesis of indeterminate potential” or CHIP is associated with a significantly elevated risk of coronary artery disease when the driving mutation is *JAK2* [4]*.* This is again in keeping with a direct effect of mutated *JAK2* on the thrombotic risk.

The complexity of management is highlighted by the use of anti-platelet, anticoagulation and cytoreduction in our cohort. Until recently, the lack of any effective agent with potential for disease modification has limited the goal of therapy to the prevention of further thrombotic events. The use of aspirin as an anti-platelet agent has been demonstrated to reduce the thrombosis risk in PV and its use has been extrapolated to high risk ET in clinical practice [18]. Anticoagulants remain the mainstay for prevention of venous thromboembolism (VTE) in the general population. We observed a heterogeneous approach to anti-platelet and anticoagulant use even in a small regional case series. There is a lack of consensus amongst haematologists treating MPN-SVT regarding anticoagulation reported [19], but it is apparent from our data that individual circumstances also impact on the therapies that are safe and tolerable. The optimum therapy for prevention of recurrent SVT and/or further VTE outside of the splanchnic veins in this setting has never been derived from a prospective clinical trial. Combination therapy using both anticoagulant and antiplatelet agents is consistently regarded as high risk for bleeding in all settings and this was evident from our observations. Oesophageal varices were common and despite concurrent management and banding under the hepatology teams, variceal haemorrhage in particular remains a significant risk. Recurrent thrombosis is also problematic. Arterial events were uncommon and only one venous thrombosis occurred on therapeutic anticoagulation during follow-up suggestive that this strategy is generally effective. The ongoing persistence of the provoking factor favours long term anticoagulation in current guidelines [20, 21]. Ongoing anticoagulation with VKA has demonstrated lower rates of thrombosis in larger series of SVT patients with MPN [22]. In a large Danish cohort studies of all cause SVT (1915 patients), higher bleeding rates have been demonstrated for up to 10 years in comparison to deep vein thrombosis or pulmonary embolus patients and for up to 19 years in comparison to the general population [23]. In another cohort of 604 all cause SVT patients including incidentally detected SVT, the rate of major bleeding was 3.9 events per 100 patient years on anticoagulation with higher rates of bleeding evident in cirrhotic patients [6]. A further cohort of 521 patients with a majority of incidental SVT diagnoses suggested that although the rates of bleeding and recurrent thrombosis were similar, the severity of bleeding complication on anticoagulation was worse [24]. The number of patients with MPN were low in all of these studies. Our study has highlighted the use of dual anti-platelet and anticoagulation results in a high bleeding risk in this specific population. Use of this strategy should therefore be carefully considered only on a case specific basis.

Direct oral anticoagulants (DOACs) have largely replaced VKA in the practical management of many thrombotic events over the past 5 years. They were not used at all in our cohort. Given the small numbers of patients involved, it is unlikely that a prospective study comparing the effectiveness of DOACS to traditional anticoagulation with VKA in the setting of *JAK2* V617F positive SVT will ever occur. One recently published treatment algorithm has suggested that their use may be justifiable in this setting [25]. This conclusion was based on the increasing demonstration of DOAC efficiency in malignancy settings and one study reporting on effective use in SVT generally [26, 27]. Individualised choice of DOACs based on the presence or absence of varices (increased gastrointestinal bleed risk with dabigatran and rivaroxaban), the extent of liver impairment and the use of potentially interacting agents may be a therapeutic strategy more widely employed in the coming years as familiarity and evidence grows with their use in atypical thrombosis sites and malignancy more generally. It is interesting to speculate how this might impact particularly on our group of individuals with both recurrent bleeding and thrombosis during follow-up.

Cytoreduction strategies have demonstrated efficacy at clot prevention in high risk patients with PV [28, 29]. During follow-up we observed a significant number of discontinuations of commonly used cytoreduction therapies including HU and interferon alpha. This was on the basis of intolerance and cytopenias. As many of these patients do not present with the classical polycythaemia or thrombocythaemia they are at risk of developing low counts as they start at a lower baseline than most MPN patients. Ruxolitinib acts as a direct JAK inhibitor and appears to be safe in this patient group [30]. In the RESPONSE studies evaluating ruxolitinib in PV, there was a reduction in thrombosis in the ruxolitinib treated arm in comparison to those patients on best available therapies [31]. Whether direct inhibition of the JAK/STAT pathway may independently reduce the risk of thrombosis in this population is a key question given the high bleeding risk that is evident, particularly in some individuals within this group. Of course ruxolitinib is also associated with cytopenias which may limit its use in this setting.

## Conclusion

The management of SVT in the setting of *JAK2* V617F positive MPN is complex. This retrospective analysis is in keeping with a younger, predominantly female cohort often with significant morbidity from liver decompensation at the outset. Bleeding rates were high during follow-up and associated with dual anticoagulation and anti-platelet administration. We did not observe recurrence of SVT outside of invasive liver intervention. Thrombosis outside of the splanchnic venous system occurred infrequently on therapeutic anticoagulation but more frequently when the patient was not on therapeutic anticoagulation. Cytoreduction is often poorly tolerated due to side effect profile or cytopenias in this group. Although we did not observe any deaths there is clearly still an unmet need to improve therapies available to this group. Understanding if direct JAK/STAT inhibition may independently reduce the risk of thrombosis is a key future step, particularly in light of the high haemorrhage risk evident.

## Abbreviations

BCS: Budd Chiari Syndrome
CHIP: Clonal Haematopoiesis of Indeterminate Potential
DOAC: Direct oral anticoagulant
DVT: Deep vein thrombosis
ET: Essential Thrombocythaemia
MPN: Myeloproliferative Neoplasm
PE: Pulmonary Embolus
PMF: Primary Myelofibrosis
PV: Polycythaemia Vera
PVT: Portal Vein Thrombosis
SVT: Splanchnic Vein Thrombosis
TIPS: Trans-jugular Intrahepatic Portosystemic Shunt
VKA: Vitamin K Antagonist
VTE: Venous thromboembolism
